# Lifestyle habits among Najran University students, Najran, Saudi Arabia

**DOI:** 10.3389/fpubh.2022.938062

**Published:** 2022-08-15

**Authors:** Awad Mohammed Al-Qahtani

**Affiliations:** Department of Family and Community Medicine, College of Medicine, Najran University, Najran, Saudi Arabia

**Keywords:** university students, dietary habits, smoking behaviors, physical activity, Saudi Arabia

## Abstract

**Background:**

Unhealthy lifestyles have been linked to increased cardiovascular disease, diabetes, and other non-communicable diseases. University students have been reported to adopt unhealthy lifestyles and undesirable eating choices.

**Objectives:**

The study aimed to estimate the prevalence of healthy eating habits; determine the prevalence of physical activity habits; to estimate the smoking habits among male health sciences college students at Najran University, Najran, Saudi Arabia.

**Methods:**

The present questionnaire-based descriptive cross-sectional study (convenient sample) was carried out among undergraduate male students enrolled in the health sciences colleges at Najran University, Najran, Saudi Arabia, from 1st May to 31st May, 2019. Around 500 students were recruited and were requested to answer a self-administered questionnaire about eating habits, physical activity, and tobacco smoking habits. Descriptive results were summarized in percentage and frequency. Cross tabulation using chi-square test was performed to measure the impact of demographic variable on eating pattern, physical activity and smoking behavior. Pearson's correlation was done using two tailed tests to determine the type of relationship between different variables. The SPSS (version 26) was used to perform statistical analysis.

**Results:**

A total of 454 complete responses from the male students from Najran University were obtained. The majority (74%) were aged 21 years and above, unmarried (94.9%), had healthy BMI (47.4%), and suffered mild (41.4%) to moderate (32.4%) levels of stress during the previous month. The study findings showed a low daily frequency of consumption of vegetables and fruits. There was a low prevalence of daily consumption of vegetables (16%) and fruits (9%). More than 10% of all students reported no consumption of fruits and vegetables. Students who felt severely stressed during the previous month were significantly not eating any vegetables (*p* = 0.022) and fruits (*p* < 0.001), and had high salt intake (*p* = 0.045). Married participants had a significantly (*p* = 0.03) higher servings of vegetables per day. Furthermore, 32.15% of participants were not practicing 30 min of physical activity 5 days per week. The study showed a low prevalence (47.57%) of physical activity among the participants. However, none of the participants' variables were significantly associated with routine physical activity. In addition, the study showed a relatively high prevalence of smoking (25.77%) among Najran university male students. The participants' age (*p* 0.01), overall health (*p* = 0.02) and level of stress (*p* = 0.001) experienced during the last month were significantly associated with the length of exposure to secondhand smoke. Whereas, smoking any kind of tobacco daily (25.77%) was significantly (*p* = 0.005) related to the age of participants. A high number of participants aged 21 years and above (52.72%) were significantly (*p* = 0.019) exposed to secondhand smoke.

**Conclusion:**

To conclude, the study findings showed a low daily frequency of consumption of vegetables and fruits, a relatively high prevalence of smoking and a low level of awareness regarding the health risks of smoking; and a large number of participants were physically inactive among male university students. Intervention programs in university students should concentrate on improving nutrition attitudes and knowledge toward good diet, tobacco-smoking cessation strategies, and structured intervention programs to encourage physical activity. However, these interventions should be pilot-tested for feasibility and acceptability before implementation.

## Introduction

In light of significant lifestyle changes, young individuals are prepared to negatively modify their eating habits regarding diversity, consumption of fruits and vegetables, and frequency and timing of intake. University years are a crucial period that can influence both the quality of lifestyle and food habits of subsequent adults and, in the long run, the health of individuals. A student's health can significantly impact high school and college academic performance. As a result, increasing the health and wellbeing of all students and faculty at a university or college requires supporting healthy eating habits and lifestyle behaviors (1–4). Overweight and obesity are common in Saudi Arabia, with prevalence rates as high as 37 and 41%, respectively. As a result, there has been an increase in nutrition-related health issues and other linked disorders because of a significant shift in dietary intake, lower physical activity levels, and a hastened nutrition transition. The Western-style diet, with its high consumption of calorie-dense foods, fats and sugars, and low fiber intake, has replaced the traditional healthy balanced diet, which included dates, milk, vegetables, fruits, fish, and whole wheat grains. Obesity is rising, especially in Saudi Arabia, because of a sedentary lifestyle and cheap and readily available junk food. These factors and dietary shifts have contributed to the problem (5).

Obesity is not just a problem for adults but also a concern for children and adolescents. Obesity and adult diseases are more likely to occur in those who are overweight or obese as children or teenagers. Obesity is most commonly seen in childhood, but university students also experience a vital period in which their behaviors are receptive to change, often increasing weight. Overweight and obese students comprise 72 of the 240 clinical students at Malaysia's International Medical University clinical students (6).

Previous research in Saudi Arabia found that university students have adopted a sedentary lifestyle, a cigarette-smoking behavior, and other unhealthy eating habits (7–9).

Students at universities make up a sizable proportion of the young adult population (10). They tend to enter an evolving transition phase of new liberation from their families, which is characterized by increased, interconnected effects on the body, mind, and social relationships (11) and discover a new atmosphere with more significant work overload, as well as altered life patterns, all of which contribute to unhealthy lifestyles. The short-term benefits of a balanced diet can boost a student's energy levels, support a healthy immune system, enhance stress management skills, and improve concentration and academic performance. The advantages of maintaining a regularly healthy diet really pile up over the course of one's life. Cancer, arthritis, memory loss, dementia, and macular degeneration are less likely to happen. There is a lower risk of developing diabetes, heart attacks, blood clots, falls and fractures, obtaining diabetes, and nutritional deficiencies that would otherwise get harder to manage as one ages. In comparison to others who have had a less healthier diet, people with a healthy diet pattern are likely to live longer, be happier, and be more active (12). A healthy eating strategy that includes more plant-based foods appears to be essential in preventing long-term diseases such as cancer, CVD, diabetes, stroke, cataracts, Alzheimer's disease, and age-related function loss (13, 14). This indicates that diet and lifestyle changes, such as boosting vegetable and fruit consumption and ensuring a much more balanced consumption of plant foods and meat, are a viable and effective strategy for reducing chronic disease incidence in the general population, especially among young students.

Physical inactivity has increased risk of cardiovascular disease, cancer, obesity, and other illnesses (15, 16). Physical activity has been shown to have numerous health benefits. Physical activity is essential to many countries' health promotion plans and international initiatives aimed at both developed and developing countries (17, 18). Previous surveys have found that adequate physical activity is comparatively higher in children and teenagers (19, 20), but considerably lower in adults, suggesting that late adolescence and early adulthood may be a critical transitional phase (21, 22). As a result, it's critical to track young adults' physical activity trends and understand characteristics like attitudes and health advantages linked to physical activity levels.

Furthermore, research indicates that students, preteens, and teenagers increasingly use many media sites and platforms. People spending more screen time are significantly associated with obesity, lesser physical activity, and health issues (23).

Smoking is a primary behavioral public health concern that affects people worldwide. It has a high yearly mortality rate, with many millions of deaths recorded yearly, and it is expected to reach ten million by 2030 (24). Like other Middle Eastern countries, Saudi Arabia is not immune to this practice, and smoking is widespread among the Saudi people. Saudi Arabia ranked eighth in an international list of countries based on their smoking prevalence (25). The number of studies on Saudi smoking behavior is limited. There is a pressing need to learn more about this phenomenon in a typical Muslim country where most residents/citizens are Muslim, and Islam forbids smoking.

Since 2005, Saudi Arabia has been a signatory to the Framework Convention on Tobacco Control (FCTC) initiative of the World Health Organization, with strict prohibitions on all forms of tobacco advertising in the national media (26). Other measures, such as prohibiting cigarette companies from sponsoring sporting events and raising tobacco taxes, were also implemented (27).

Smoking is prohibited in the kingdom's workplaces, governmental institutions, public transportation, cultural institutions, and banks (28). There are no research results available in Saudi Arabia that illustrate the legal measures' influence on the Saudi people's smoking behavior. The existing smoking research results were based on descriptive studies that focused on smoking prevalence without any follow-up on smoking practices or an emphasis on quitting (29). The demographic and social situation can significantly influence college students in the kingdom to start smoking. Students are transitioning from a more conservative parental setting with stricter controls to a more open social atmosphere at university. To better restrict the spread of smoking among the Saudi populace, governmental authorities established legislation prohibiting smoking in all public places, including educational institutions.

The Saudi Arabian National Education Policy recognizes the connection between academic achievement and student health. The following are some of its directives: (1) Students should begin school with the healthy psychological, physical, and mental alertness required for learning; (2) A safe and disciplined school environment is required to ensure students' psychological and physical well-being; (3) A chance for all students to learn healthy living skills and receive health education. (4) In the school setting, the student's development and growth “psychosocially, mentally, and physically” should be maintained in conditions similar to those governed by Islamic teachings at home. (5) All students should have access to free health care (both preventative and curative) (30).

Today's students are the future citizens of a nation. As a result, their protection, survival, and development are essential for developing the country's future. National development should prioritize providing knowledge and resources to the younger generation to meet their fundamental imperatives and pursue excellence. Children's health, nutrition, and education are critical for national development because their individual growth and social contributions will shape the country's future and assist in achieving the Saudi Vision-2030's primary goals.

Thus, the objectives of this study were as follows: (1) to estimate the prevalence of plant based eating habits (fruits and vegetables); (2) to determine the prevalence of routine physical activity; (3) to estimate the smoking habits among male health sciences students at Najran University, Najran, Saudi Arabia. The findings of this study will aid policymakers in making decisions and encouraging schools to embrace and maintain proper healthcare programs, and addressing problems that may occur during the implementation of education and health programs at the national and regional levels.

## Methodology

### Study design and participants

The present questionnaire-based descriptive cross-sectional study was carried out among undergraduate male students enrolled in the health sciences colleges at Najran University, Najran, Saudi Arabia, from 1st May to 31st May, 2019. Male students who were free of diet-related health problems, did not follow any special diet, and consumed the usual mixed diet were included. Students from other universities, graduated and students from non-health sciences programs were excluded from the participation. Study purpose and informed consent were obtained from all participants before filling of paper-based questionnaires. Participation in this study was completely voluntary. The study was approved by the ethical committee of Najran University (NU/2019/12/3).

### Sample size and sampling technique

A total of 500 male students (Approx.) are enrolled in the health sciences colleges in Najran University. Sample size was calculated using Raosoft sample size (http://www.raosoft.com/samplesize.html) calculator by presuming a 95% confidence level, 5% margin of error, and response distribution of 50%, yielding a sample size of 218. We adopted convenience sampling techniques to collect the data. However, to represent the better response rate, and assuming few incomplete and inconsistent responses, the total samples collected were 454.

### Study tool and validation

The detailed review of relevant literature was carried out to develop the initial questionnaire draft (31–40). Study tool was validated for its content from experts in the field of community health, dieticians, and pharmacist. Some of the items from questionnaire were removed from the initial draft based on recommendations from these experts. These questionnaires were tested for the face validity among 25 male undergraduate students of Najran University. The reliability of found to be satisfactory upon calculating alpha Cronbach factor (0.76). The final questionnaires were categorized into following sections: section one noted participants' demographic details such as age, marital status, BMI, presence of chronic disease, perceived level of stress experienced in last month (No, mild, moderate and severe) and self-declared overall health status. We collected the Height (cm) and weight (kg) from students to calculate the BMI. The BMI was categorized to underweight (<18.5), healthy (18.5 to <25), overweight (25.0 to <30) and obese (30.0 or higher) using BMI index chart from CDC (The Centers for Disease Control and Prevention). Second section was designed to record the pattern (Do you eat fruits daily) and frequency (how many pieces of fruits you eat per day) of consumption of vegetables, fruits and addition of salt to regular food among study participants. Third section explored the engagement of students in routine physical activity of 30 mins or above at least 5 days per week, followed by perception about benefits of performing routine physical activity and factor preventing them from exercising. The last section noted students smoking pattern (do you smoke daily), exposure to secondhand smoke, diseases caused by active and passive smoking and opinion about harmful effects of smoking.

### Data collection

The paper-based questionnaire was distributed to all the participants at the entrance of university campus. The study purpose, eligibility criteria was briefed to every participant. Students were ensured about the confidentiality of their identity. A designated area with sitting arrangement and writing material was arranged for the participants to complete the questionnaire without any disturbances. Participants were encouraged to complete all the items of survey. The liker scale (Strongly agree to strongly disagree) was used to explore the students perception about benefits of daily routine exercise. Awareness about negative impact of active and passive smoking was documented using yes, no and don't options.

### Statistical analysis

All the collected responses were checked for their completeness and consistency. Pilot study, incomplete and repeat responses were excluded from the final data analysis. Then responses were entered into Microsoft excel sheets and coded before subjecting them for analysis. The SPSS (version 26) was used to perform statistical analysis. Results were summarized in percentage, frequency, tabular and graphical forms. Cross tabulation using chi-square test was performed to measure the impact of demographic variable on eating pattern, physical activity and smoking behavior. Pearson's correlation was executed (using two tailed *p* value tests at a significance of <0.05 and 0.01) to determine the type of relationship between addition of salt to regular meal, physical activity, smoking of tobacco with opinions about harmful effects of smoking.

## Results

### Participants' details

A total of 454 male students from Najran University participated in this study. Majority (74%) of respondents were aged 21 years and above, unmarried (94.9%), having healthy BMI (47.4%). More than half of students have suffered mild (41.4%) to moderate (32.4%) level of stress during last month. About half of students have self-reported their overall health condition as good (21.8%) and very good (38.1%) as depicted in Table 1.

**Table 1:** Socio-demographic characteristics of the study participants (*n* = 454).

**Demographic variables**		**Frequency**	**Percentage (%)**
Age	20 years and below	118	26.0
	21 years and above	336	74.0
Marital Status	Single	431	94.9
	Married	22	4.8
	Divorced/separated	1	0.2
BMI	Underweight	47	10.4
	Healthy	215	47.4
	Overweight	115	25.3
	Obese	77	17.0
Do you suffer from chronic disease	Yes	61	13.4
	No	393	86.6
Did you feel stressed last month (Percieved level of stress)?	No	77	17.0
	Mild	188	41.4
	Moderate	147	32.4
	Severe	42	9.3
How to you rate your overall health	Very bad	7	1.5
	Bad	28	6.2
	Average	89	19.6
	Good	99	21.8
	Very good	173	38.1
	Excellent	58	12.8

### Vegetable, fruits eating pattern and adding salt to food among male students of Najran University

Addition of salt, consumption of vegetables and fruits weekly was not significantly associated with the participants' age, marital status, BMI, presence of chronic disease and self-declared overall health status. However, the stress felt during the last month had significantly affected addition of salt to food (*p* = 0.045), vegetable consumption per week (*p* = 0.022) and fruits consumption per week (*p* < 0.001). The participants with severe (76.2%) and moderate (69.4%) stress have significantly added salt to their food always compared to participants with no or mild stress. Similarly, consumption of vegetables and fruits was significantly low among participants who felt stressed during last month. About 21.4 and 23.8% of participants who felt severely stressed during last month were significantly not eating any vegetables and fruits respectively compared to the students who were not stressed and mildly stressed (Table 2).

**Table 2:** Vegetable, fruits eating pattern and adding salt to food among male students of Najran University.

**Demographic variables**		**Do you add salt to your food**				**Usually how many days a week do you eat vegetables**					**Usually how many days a week do you eat fruits**				
		**Always**	**Sometimes**	**No**	***p*** **value**	**No**	**1–3 days**	**4–6 days**	**Everyday**	***p*** **value**	**No**	**1–3 days**	**4–6 days**	**Everyday**	***p*** **value**
Age	20 years and below	88 (74.6%)	26 (22%)	4 (3.4%)	0.115	15 (12.7%)	55 (46.6%)	33 (28%)	15 (12.7%)	0.581	11 (9.3)	67 (56.8%)	27 (22.9%)	13 (11%)	0.520
	21 years and above	217 (64.6%)	108 (32.1%)	11 (3.3%)		33 (9.8%)	158 (47%)	87 (25.9%)	58 (17.3%)		35 (10.4%)	176 (52.4%)	97 (28.9%)	28 (8.3%)	
Marital status	Single	287 (66.6%)	131 (30.4%)	13 (3%)	0.270	46 (10.7%)	201 (46.6%)	117 (27.1%)	67 (15.5%)	0.563	44 (10.2%)	228 (52.9%)	120 (27.8%)	39 (9%)	0.867
	Married	17 (77.3%)	3 (13.6%)	2 (9.1%)		2 (9.1%)	11 (50%)	3 (13.6%)	6 (27.2%)		2 (9.1%)	14 (63.6%)	4 (18.2%)	2 (9.1%)	
	Divorced/separated	1 (100%)	Zero	Zero		Zero	1 (100%)	Zero	Zero		Zero	1 (100%)	Zero	Zero	
BMI	Underweight	30 (63.8%)	15 (31.9%)	2 (4.3%)	0.747	5 (10.6%)	21 (447%)	12 (25.5%)	9 (19.1%)	0.978	2 (4.3%)	31 (66%)	11 (23.4%)	3 (6.4%)	0.100
	Healthy	147 (68.4%)	61 (28.4%)	7 (3.3%)		23 (10.7%)	102 (47.4%)	56 (26.1%)	34 (15.8%)		20 (9.3)	114 (53%)	58 (27%)	23 (10.7%)	
	Overweight	72 (62.6%)	40 (34.8%)	3 (2.6%)		11 (9.6%)	58 (50.4%)	31 (27%)	15 (13%)		16 (13.9%)	55(47.8%)	39 (33.9%)	5 (4.4%)	
	Obese	56 (72.7%)	18 (23.4%)	3 (3.9%)		9 (11.7%)	32 (41.6%)	21 (27.3%)	15 (19.5%)		8 (10.4%)	43 (55.8%)	16 (20.8%)	10 (13%)	
Do you suffer from Chronic disease	Yes	41 (67.2%)	19 (31.2%)	1 (1.6%)	0.681	4 (6.6%)	27 (44.3%)	16 (26.2%)	14 (23%)	0.363	4 (6.6%)	34 (55.7%)	13 (21.3%)	10 (16.4%)	0.133
	No	264 (67.2%)	115 (29.3%)	14 (3.6%)		44 (11.2%)	186 (47.3%)	104 (26.5%)	59 (15%)		42 (10.7%)	209 (53.2%)	111 (28.2%)	31 (7.9%)	
Did you feel stressed last month?	No	48 (62.3%)	26 (33.8%)	3 (3.9%)	0.045^*^	6 (7.8%)	26 (33.8%)	30 (39%)	15 (19.5%)	0.022^*^	6 (7.8%)	30 (39%)	30 (39%)	11 (14.3%)	<0.001^*^
	Mild	123 (65.4%)	54 (28.7%)	11 (5.9%)		19 (10.1%)	91 (48.4%)	45 (23.9%)	33 (17.6%)		10 (5.3%)	108 (57.5%)	52 (27.7%)	18 (9.6%)	
	Moderate	102 (69.4%)	44 (29.9%)	1 (0.7%)		14 (9.5%)	80 (54.4%)	36 (24.5%)	17 (11.6%)		20 (13.6%)	88 (59.9%)	32 (21.8%)	7 (4.8%)	
	Severe	32 (76.2%)	10 (23.8%)	Zero		9 (21.4%)	16 (38.1%)	9 (21.4%)	8 (19.1%)		10 (23.8%)	17 (40.5%)	10 (23.8%)	5 (11.9%)	
How to you rate your overall health	Very bad	7 (100%)	Zero	Zero	0.199	4 (57.1%)	3 (42.9%)	Zero	Zero	0.039	2 (28.6%)	2 (28.6%)	3 (42.9%)	Zero	0.144
	Bad	21 (75%)	7 (25%)	Zero		5 (17.9%)	14 (50%)	6 (21.4%)	3 (10.7%)		4 (14.3%)	16 (57.1%)	6 (21.4%)	2 (7.1%)	
	Average	56 (62.9%)	29 (32.6%)	4 (4.5%)		7 (7.9%)	42 (47.2%)	22 (24.7%)	18 (20.2%)		8 (9%)	46 (51.7%)	24 (27%)	11 (12.4%)	
	Good	62 (62.6%)	31 (31.3%)	6 (6.1%)		12 (12.1%)	42 (42.4%)	35 (35.4%)	10 (10.1%)		9 (9.1%)	62 (62.6%)	26 (26.3%)	2 (2%)	
	Very good	117 (67.6%)	53 (30.6%)	3 (1.7%)		15 (8.7%)	81 (46.8%)	46 (26.6%)	31 (17.9%)		19 (11%)	85 (49.1%)	52 (30.1%)	17 (9.8%)	
	Excellent	42 (72.4%)	14 (24.1%)	2 (3.5%)		5 (8.6%)	31 (53.5%)	11 (19%)	11 (19%)		4 (6.9%)	32 (55.2%)	13 (22.4%)	9 (15.5%)	

* p value < 0.05.

### Pattern of consumption of fruits and vegetables per day among male students of Najran University

In this section, we evaluated the student's pattern and frequency of consumption of vegetables and fruits per day. Eating pattern of fruits and vegetables was not significantly associated with any of the participants' variable except level of stress felt during last month. Participants who experienced severe stress in last month were significantly (*p* = 0.002) higher (31%) in not eating any fruit pieces per day compared to the ones who didn't experience ay stress or felt mild stress (Table 3).

**Table 3:** Pattern of consumption of fruits and vegetables per day among male students of Najran University.

**Demographic variables**		**How many pieces of fruit do you eat per day**			***p*** **value**	**Usually how many servings of vegetables do you eat per day**			***p*** **value**
		**Don't eat**	**1–4 pieces**	**5 and more pieces**		**Don't eat**	**1–4 serving**	**5 and more serving**	
Age	20 years and below	17 (14.4%)	94 (79.7%)	7 (5.9%)	0.751	15 (12.7%)	92 (78%)	11 (9.3%)	0.16
	21 years and above	40 (11.9%)	273 (81.3%)	23 (6.9%)		25 (7.4%)	269 (80.1%)	42 (12.5%)	
Marital status	Single	54 (12.5%)	349 (81%)	28 (6.5%)	0.229	40 (9.3%)	344 (79.8%)	47 (10.9%)	0.03
	Married	3 (13.6%)	18 (81.8%)	1 (4.6%)		Zero	17 (77.3%)	5 (22.7%)	
	Divorced/ separated	Zero	Zero	1 (100%)		Zero	Zero	1(100%)	
BMI	Underweight	6 (12.8%)	39 (83%)	2 (4.3%)	0.620	5 (10.6%)	41 (87.2%)	1 (2.1%)	0.10
	Healthy	21 (9.8%)	178 (82.8%)	16 (7.4%)		16 (7.4%)	177 (82.3%)	22 (10.2%)	
	Overweight	19 (16.5%)	90 (78.3%)	6 (5.2%)		11 (9.6%)	87 (75.7%)	17 (14.8%)	
	Obese	11 (14.3%)	60 (77.9%)	6 (7.8%)		8 (10.4%)	56 (72.7%)	13 (16.9%)	
Do you suffer from chronic disease	Yes	7 (11.5%)	48 (78.7%)	6 (9.8%)	0.544	5 (8.2%)	44 (72.1%)	12 (19.7%)	0.11
	No	50 (12.7%)	319 (81.2%)	24 (6.1%)		35 (8.9%)	317 (80.7%)	41 (10.4%)	
Did you feel stressed last month?	No	8 (10.4%)	63 (81.8%)	6 (7.8%)	0.002^*^	5 (6.5%)	63 (81.8%)	9 (11.7%)	0.25
	Mild	13 (6.9%)	166 (88.3%)	9 (4.8%)		15 (8%)	150 (79.8%)	23 (12.2%)	
	Moderate	23 (15.7%)	113 (76.9%)	11 (7.5%)		11 (7.5%)	118 (80.3%)	18 (12.2%)	
	Severe	13 (31%)	25 (59.5%)	4 (9.5%)		9 (21.4%)	30 (71.4%)	3 (7.1%)	
How to you rate your overall health	Very bad	2 (28.6%)	4 (57.1%)	1 (14.3%)	0.667	1 (14.3%)	6 (85.7%)	Zero	0.76
	Bad	6 (21.4%)	21 (75%)	1 (3.6%)		4 (14.3%)	22 (78.6%)	2 (7.1%)	
	Average	12 (13.5%)	71 (79.8%)	6 (6.7%)		9 (10.1%)	70 (78.7%)	10 (11.2%)	
	Good	10 (10.1%)	84 (84.9%)	5 (5.1%)		8 (8.1%)	81 (81.8%)	10 (10.1%)	
	Very good	20 (11.6%)	138 (79.8%)	15 (8.7%)		11 (6.4%)	139 (80.4%)	23 (13.3%)	
	Excellent	7 (12.1%)	49 (84.5%)	2 (3.5%)		7 (12.1%)	43 (74.1%)	8 (13.8%)	

* P value < 0.05.

### Engagement of daily 30 mins' physical activity among male students of Najran University

This section evaluated the duration of student's engagement in performing routine physical activity of 30 mins or above for at least 5 days a week. We observed that nearly half of students (47.5%) were performing daily 30 mins physical activity. None of the participants' variables were significantly associated with routine physical activity. However, overweight students were non-significantly higher (31%) in performing routine physical activity of 30 mins per day for since more than 6 months compared to underweight, healthy ad obese students. Likewise, students who were planning to start routine physical activity were higher number (26%) from obese category. Students who felt severely stressed ding last month, were non-significant higher (14.3%) in not performing any physical activity compared to their counterparts (Table 4).

**Table 4:** Engagement into daily 30 mins physical activity 5 days/week among male students of Najran University.

**Demographic variables**		**Duration of daily 30 mins physical activity 5 days/week**					***p*** **value**
		**Never**	**Not in last 6 months**	**I'm planning to involve**	**Yes, but less than 6 months**	**Yes, more than 6 months**	
Age	20 years and below	9 (7.6%)	35 (29.7%)	23 (19.5%)	33 (28%)	18 (15.3%)	0.52
	21 years and above	29 (8.6%)	73 (21.7%)	69 (20.5%)	103 (30.7%)	62 (18.5%)	
Marital status	Single	36 (8.4%)	103 (23.9%)	86 (20%)	128 (29.7%)	78 (18.1%)	0.84
	Married	2 (9.1%)	5 (22.7%)	6 (27.3%)	7 (31.8%)	2 (9.1%)	
	Divorced/separated	Zero	Zero	Zero	1 (100%)	Zero	
BMI	Underweight	6 (12.8%)	10 (21.3%)	12 (25.5%)	14 (29.8%)	5 (10.6%)	0.10
	Healthy	16 (7.4%)	45 (20.9%)	32 (14.9%)	73 (34%)	49 (22.8%)	
	Overweight	10 (8.7%)	31 (27%)	28 (24.4%)	31 (27%)	15 (31%)	
	Obese	6 (7.8%)	22 (28.6%)	20 (26%)	18 (23.4%)	11 (14.3%)	
Do you suffer from Chronic disease	Yes	8 (13.1%)	14 (23%)	12 (19.7%)	18 (29.5%)	9 (14.8%)	0.68
	No	30 (7.6%)	94 (23.9%)	80 (20.4%)	118 (30%)	71 (18.1%)	
Did you feel stressed last month?	No	7 (9.1%)	23 (29.9%)	12 (15.6%)	21 (27.3%)	14 (18.2%)	0.54
	Mild	14 (7.5%)	37 (19.7%)	35 (18.6%)	66 (35.1%)	36 (19.2%)	
	Moderate	11 (7.5%)	38 (25.9%)	35 (23.8%)	39 (26.5%)	24 (16.3%)	
	Severe	6 (14.3%)	10 (23.8%)	10 (23.8%)	10 (23.8%)	6 (14.3%)	
How to you rate your overall health	Very bad	Zero	5 (71.4%)	Zero	2 (28.6%)	Zero	0.43
	Bad	5 (17.9%)	7 (25%)	5 (17.9%)	8 (28.6%)	3 (10.7%)	
	Average	7 (7.9%)	16 (18%)	21 (23.6%)	27 (30.3%)	18 (20.2%)	
	Good	8 (8.1%)	23 (23.2%)	21 (21.2%)	34 (34.3%)	13 (13.1%)	
	Very good	15 (8.7%)	42 (24.3%)	34 (19.7%)	47 (27.2%)	35 (20.2%)	
	Excellent	3 (5.2%)	15 (25.9%)	11 (19%)	18 (31%)	11 (19%)	

### Perception about the benefits of performing 30 mins of regular physical activity or exercise for 5 days/week

**Figure 1:**
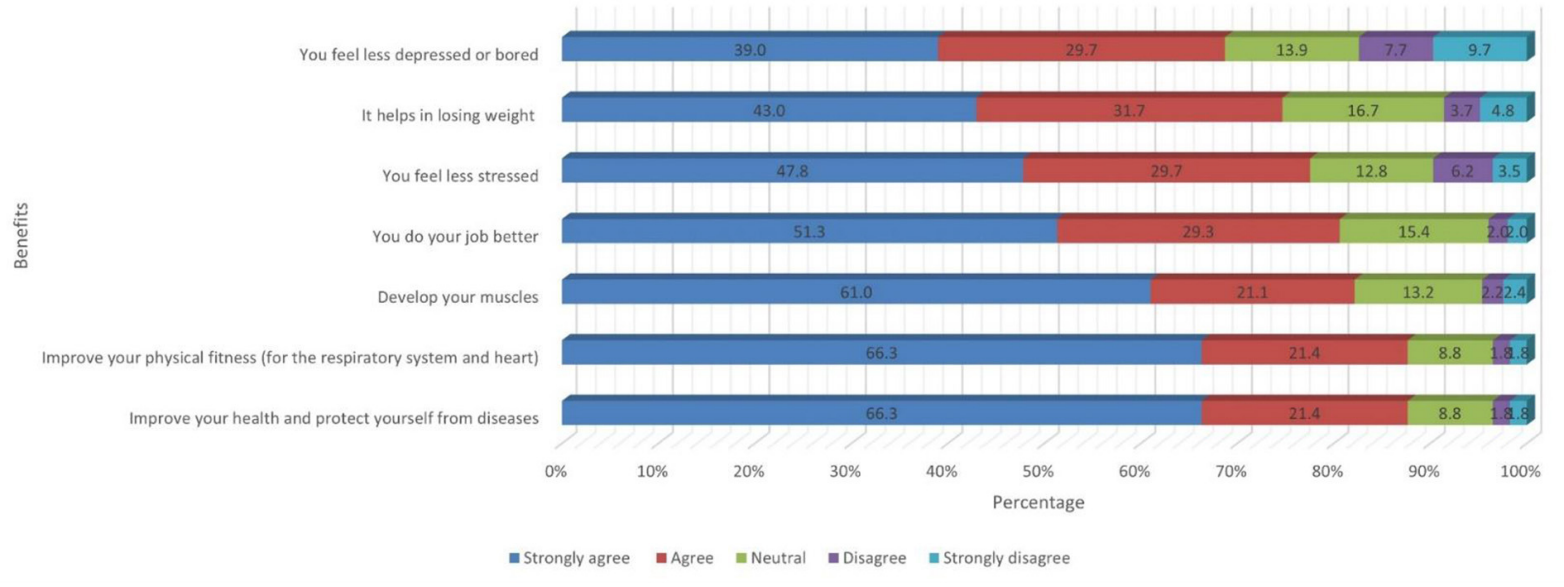
Perception about the benefits of performing 30 mins of regular physical activity or exercise for 5 days/week.

This domain assessed the student's perception about the benefits of performing routine physical activity. More than 80% of students believe (strongly agree and agree) that performing 30 mins of regular physical activity or exercise for 5 days/week, would improve health and protect from diseases (87.7%), improve physical fitness (87.7%), develop body muscles (82.1%) and helps in doing better job (80.6%). Impact of physical activity on psychological health was noted using two statements. About 77.5% and 68.7% of students believe (strongly agree and agree) that performing routine physical activity of 30 mins will make the person feel less stressed and depressed respectively. However, 8.5% of students disagree that routine physical activity was not impact on losing body weight (Figure 1).

### Factors preventing exercising among students

**Figure 2:**
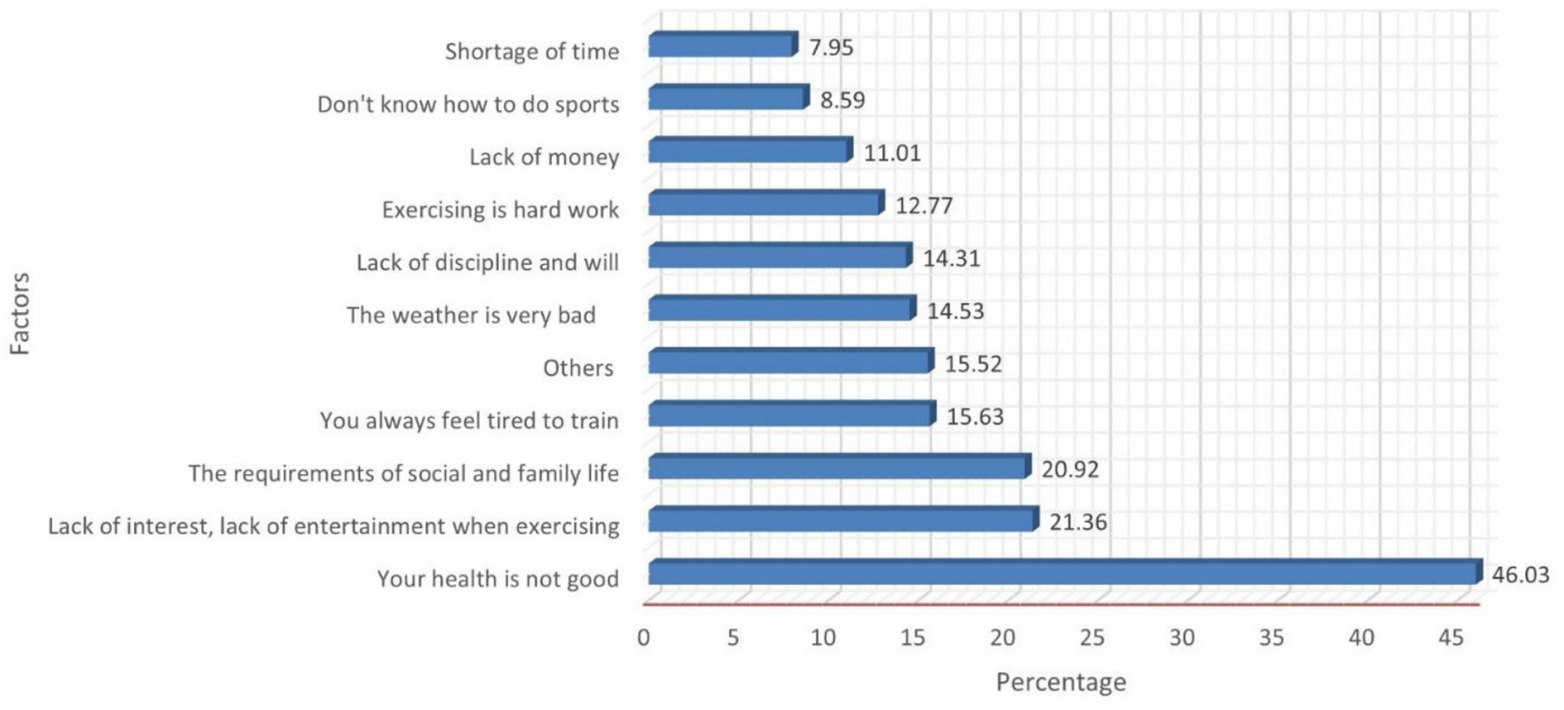
Factors preventing from exercising.

Students mentioned some of the important factors which discourage them from performing routine physical activity or exercise. Nearly, half of students (46.03%) of students stated weak health as most common discouraging factor, followed by lack of interest and entertainment (21.36%), busy with social and family life (20.92%) and feeling of tiredness (15.63%). The other (work requirement, fear of harm, obese and pain) reasons preventing from regular exercise accounted for 15.52%. The least common factor mentioned were shortage of time (7.95%), lack of money (11%) and believing that exercise is hard work (12.77%) (Figure 2).

### Tobacco smoking and exposure to secondhand smoke among male students of Najran University

The nature of smoking and exposure to secondhand smoke was noted in this section. The participants' age, overall health and level of stress experienced during last month was significantly associated with the length of exposure to secondhand smoke. Whereas smoking any kind of tobacco on daily basis was significantly related to age of participants. High number of participants aged 21 years and above were significantly (*p* = 0.019) exposed to secondhand smoke. Exposure to secondhand smoke daily for 1–20 mins (11.9%) and 21–60 mins (15.2%) was higher among students aged 21 years and above, which is twice the students aged below 20 years (6.8% and 8.5%). Students aged 21 years and above were significantly (*p* = 0.005) higher (29.2%) in smoking any kind of tobacco compared to younger students (16.1). Moreover, older students were significantly (*p* = 0.031) high (25.9%) in daily smoking compared to their younger counterparts (16.1%). Participants wo experienced severe stress durn last month were significantly (*p* < 0.001) highly exposed to secondhand smoke for 1–20 mins (23.8%) and 21–60 mins (23.8%). Students who stated their health as very bad (42.9%) and bad (21.4%) were significantly (*p* = 0.029) highly exposed to secondhand smoke for 1–20 and 21–60 mins respectively. Other variables didn't affect the smoking pattern and exposure to secondhand smoke significantly (Table 5).

**Table 5:** Tobacco smoking and exposure to secondhand smoke among male students of Najran University.

**Demographic variables**		**On a typical day, how long are you exposed to secondhand smoke**				***p*** **value**	**Currently, do you smoke any type of tobacco**		***p*** **value**	**Do you currently smoke daily**		***p*** **value**
		**No**	**1–20 min**	**21–60 min**	**More than 60 min**		**Yes**	**No**		**Yes**	**No**	
Age	20 years and below	94 (79.7%)	8 (6.8%)	10 (8.5%)	6 (5.1%)	0.01^*^	19 (16.1%)	99 (83.9%)	0.005^*^	19 (16.1%)	99 (83.9%)	0.03^*^
	21 years and above	220 (65.5%)	40 (11.9%)	51 (15.2%)	25 (7.4%)		98 (29.2%)	238 (70.8%)		87 (25.9%)	249 (74.1%)	
Marital status	Single	300 (69.6%)	47 (10.9%)	58 (13.5%)	26 (6%)	0.44	108 (25.1%)	323 (74.9%)	0.212	98 (22.7%)	333 (77.3%)	0.28
	Married	13 (59.1%)	1 (4.6%)	3 (13.6%)	5 (22.7%)		9 (40.9%)	13 (59.1%)		8 (36.4%)	14 (63.6%)	
	Divorced/separated	1 (100%)	0	0	0		0	1 (100%)		0	1 (100%)	
BMI	Underweight	31 (66%)	9 (19.2%)	3 (6.4%)	4 (8.5%)	0.24	15 (31.9%)	32 (68.1%)	0.402	12 (25.5%)	35 (74.5%)	0.45
	Healthy	158 (73.5%)	19 (8.8%)	28 (13%)	10 (4.7%)		50 (23.3%)	165 (76.7%)		47 (21.9%)	168 (78.1%)	
	Overweight	79 (68.7%)	10 (8.7%)	15 (13%)	11 (9.6%)		28 (24.4%)	87 (75.7%)		24 (20.9%)	91 (79.1%)	
	Obese	46 (59.7%)	10 (13%)	15 (19.5%)	6 (7.8%)		24 (31.2%)	53 (68.8%)		23 (29.9%)	54 (70.1%)	
Do you suffer from chronic disease	Yes	42 (68.9%)	5 (8.2%)	8 (13.1%)	6 (9.8%)	0.79	15 (24.6%)	46 (75.4%)	0.821	14 (23%)	47 (77.1%)	0.93
	No	272 (69.2%)	43 (10.9%)	53 (13.5%)	24 (6.1%)		102 (26%)	291 (74.1%)		92 (23.4%)	301 (76.6%)	
Did you feel stressed last month?	No	62 (80.5%)	1 (1.3%)	11 (14.3%)	3 (3.9%)	0.001^*^	15 (19.5%)	62 (80.5%)	0.155	16 (20.8%)	61 (79.2%)	0.69
	Mild	147 (78.2%)	17 (9%)	14 (7.5%)	10 (5.3%)		44 (23.4%)	144 (76.6%)		41 (21.8%)	147 (78.2%)	
	Moderate	87 (59.2%)	20 (13.6%)	26 (17.7%)	14 (9.5%)		43 (29.3%)	104 (70.8%)		37 (25.2%)	110 (74.8%)	
	Severe	18 (42.9%)	10 (23.8%)	10 (23.8%)	4 (9.5%)		15 (35.7%)	27 (64.3%)		12 (28.6%)	30 (71.4%)	
How to you rate your overall health	Very bad	3 (42.9%)	3 (42.9%)	1 (14.3%)	Zero	0.029^*^	2 (28.6%)	5 (71.4%)	0.887	1 (14.3%)	6 (85.7%)	0.91
	Bad	14 (50%)	5 (17.9%)	6 (21.4%)	3 (10.7%)		9 (32.1%)	19 (67.9%)		8 (28.6%)	20 (71.4%)	
	Average	68 (76.4%)	7 (7.9%)	8 (9%)	6 (6.7%)		23 (25.8%)	66 (74.2%)		21 (23.6%)	68 (76.4%)	
	Good	65 (65.7%)	16 (16.2%)	10 (10.1%)	8 (9.1%)		24 (24.2%)	75 (75.8%)		24 (24.2%)	75 (75.8%)	
	Very good	114 (65.9%)	15 (8.7%)	33 (19.1%)	11 (6.4%)		47 (27.2%)	126 (72.8%)		41 (23.7%)	132 (76.3%)	
	Excellent	50 (86.2%)	2 (3.5%)	3 (5.2%)	3 (5.2%)		12 (20.7%)	46 (79.3%)		11 (19%)	47 (81%)	

* p value < 0.05.

### Student's belief about the diseases caused by active and passive smoking

**Figure 3:**
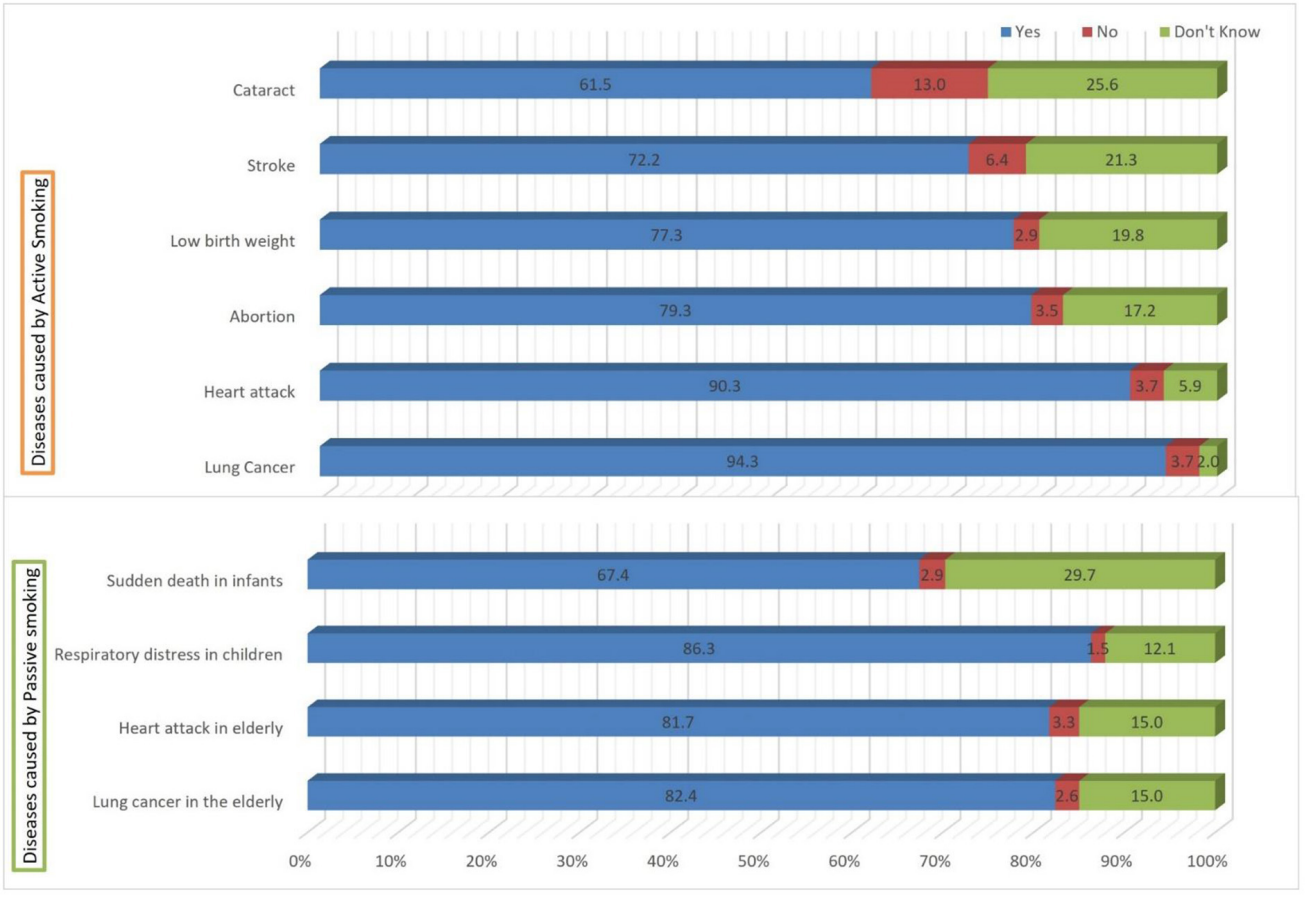
Student's belief about the diseases caused by active and passive smoking.

More than 90% of students believe that active smoking causes lung cancer (94.3%) and heart attack (90.3%) followed by abortion (79.3%) and low birth weight (77.3%) in pregnant females and stroke (72.2%). Whereas, about 61.5% students mentioned active smoking could also lead to cataract. Likewise, participants stated their agreement with the diseases caused by passive smoking. Majority of participants believe that passive smoking causes lung cancer (82.4%) and heart attack (81.7%) in elderly people followed by respiratory distress in children (86.3%) and sudden death in infants (67.4%) as depicted in Figure 3.

### Correlation between opinions with smoking, physical activity, and salt addition to food

Bivariate Pearson analysis was performed to find out the correlation and its nature among various variables (Table 6). The analysis revealed significant (*p* = 0.039) positive correlation (r = 0.097) between addition of salt to food with 30 mins or more of physical activity or exercise 5 days per week, indicating that those who add salt to their food are most likely performing routine physical activity. Likewise, tobacco smoking was significantly positively associated with daily smoking (r = 0.865, *p* < 0.001), opinion 2 (smoking is addictive, r = 0.163, *p* < 0.001) and opinion 3 (smoking shisha is harmful to health, r = 0.133, *p* = 0.005). This indicates that those who smoke any form of tobacco (cigarette, cigar, hookah, pipe) are more likely to smoke daily and agree with the opinion 2 and 3. A significant positive correlation was observed among the opinions. Whereas opinion 1 (light cigarettes are less harmful than regular cigarettes) was non-significantly negatively related to addition of salt to food, routine physical activity, tobacco smoking and daily smoking.

**Table 6:** Pearson correlation between opinions with smoking, physical activity, and salt addition to food.

**Variable**	**Adding salt to food**		**Physical activity 5 days a week**		**Smoking tobacco**		**Daily smoking**		**Opinion 1**		**Opinion 2**		**Opinion 3**	
	**r**	***p*** **value**	**r**	***p*** **value**	**r**	***p*** **value**	**r**	***p*** **value**	**r**	***p*** **value**	**r**	***p*** **value**	**r**	***p*** **value**
Adding salt to food	–	–	0.097^*^	0.039	0.030	0.521	0.041	0.383	−0.017	0.715	0.037	0.435	0.002	0.973
Physical activity 5 days a week			–	–	0.049	0.302	0.085	0.070	−0.022	0.647	0.007	0.879	0.019	0.688
Smoking tobacco					–	–	0.865^**^	<0.001	−0.014	0.771	0.163^**^	<0.001	0.133^**^	0.005
Daily smoking							–	–	−0.034	0.464	0.159^**^	0.001	0.130^**^	0.006
Opinion 1									–	–	0.162^**^	0.001	0.108^*^	0.021
Opinion 2											–	–	0.642^**^	<0.001

r = correlation coefficient.

* Significant correlation at the 0.05 level (2-tailed).

** Significant correlation at the 0.01 level (2-tailed).

Opinion 1: Light cigarettes are less harmful than regular cigarettes.

Opinion 2: Smoking is addictive.

Opinion 3: Smoking shisha is harmful to health.

### Opinion regarding the harmful effects of smoking

Three statements were used to record the participants' opinion toward harmful effects of smoking. We noted a significant (*p* = 0.036) impact of marital status toward harmful effect of light cigarette compared to regular cigarettes. Unlike unmarried students, married students (40.9%) disagree that “Light cigarettes are less harmful than regular cigarettes.” Conversely, more than 80% of students agree that smoking is addictive and smoking shisha is harmful to health. No significant impact of marital status was observed on the addictive nature of smoking and harmful effects of shisha smoking (Table 7). We noted significant (*p* = 0.05) impact of health status on the harmful effect of light cigarette compared to regular cigarettes. About 40% of students with bad health status disagree that “Light cigarettes are less harmful than regular cigarettes,” whereas, students with good and excellent health agree with this. No significant impact of health status was noted on addictive nature of smoking and harmful effects of shisha smoking.

**Table 7:** Opinion regarding the harmful effects of smoking.

**Statement**	**Opinions**	**Marital Status**			***p*** **value**	**How do you rate your overall health (Health status)**						***p*** **value**
		**Single**	**Married**	**Widowed/ Separated**		**Very bad**	**Bad**	**Average**	**Good**	**Very good**	**Excellent**	
Light cigarettes are less harmful than regular cigarettes	Strongly agree	134 (31.1%)	2 (9.1%)	1 (100%)	0.036^*^	Zero	8 (28.6%)	18 (20.2%)	33 (33.3%)	51 (29.5%)	27 (46.6%)	0.005^*^
	Agree	152 (35.3%)	5 (22.7%)	Zero		6 (85.7%)	5 (17.9%)	40 (44.9%)	29 (29.3%)	64 (37%)	13 (22.4%)	
	Neutral	42 (9.7%)	6 (27.3%)	Zero		Zero	4 (14.3%)	11 (12.4%)	11 (11.1%)	18 (10.4%)	4 (6.9%)	
	Disagree	25 (5.8%)	1 (4.5%)	Zero		Zero	1 (3.6%)	6 (6.7%)	2 (2%)	12 (6.9%)	5 (8.6%)	
	Strongly Disagree	78 (18.1%)	8 (36.4%)	Zero		1 (14.3%)	10 (35.7%)	14 (15.7%)	24 (24.2%)	28 (16.2%)	9 (15.5%)	
Smoking is addictive	Strongly agree	205 (47.6%)	13(59.1%)	1 (100%)	0.427	2 (28.6%)	15 (53.6%)	40 (44.9%)	49 (49.5%)	77 (44.5%)	36 (62.1%)	0.33
	Agree	172 (39.9%)	7 (31.8%)	Zero		5 (71.4%)	7 (25%)	39 (43.8%)	40 (40.4%)	71 (41%)	17 (29.3%)	
	Neutral	24 (5.6%)	Zero	Zero		Zero	3 (10.7%)	3 (3.4%)	5 (5.1%)	12 (6.9%)	1 (1.7%)	
	Disagree	13 (3%)	2 (9.1%)	Zero		Zero	Zero	3 (3.4%)	2 (2%)	7 (4%)	3 (5.2%)	
	Strongly Disagree	17 (3.9%)	Zero	Zero		Zero	3 (10.7%)	4 (4.5%)	3 (3%)	6 (3.5%)	1 (1.7%)	
Smoking shisha is harmful to health	Strongly agree	269 (62.4%)	13 (59.1%)	1 (100%)	0.691	3 (42.9%)	17 (60.7%)	56 (62.9%)	65 (65.7%)	100 (57.8%)	42 (72.4%)	0.08
	Agree	127 (29.5%)	5 (22.7%)	Zero		3 (42.9%)	5 (17.9%)	28 (31.5%)	27 (27.3%)	56 (32.4%)	13 (22.4%)	
	Neutral	17 (3.9%)	3 (13.6%)	Zero		1 (14.2%)	3 (10.7%)	2 (2.2%)	6 (6.1%)	8 (4.6%)	Zero	
	Disagree	9 (2.1%)	Zero	Zero		Zero	Zero	1 (1.1%)	1 (1%)	6 (3.5%)	1 (1.7%)	
	Strongly disagree	9 (2.1%)	1 (4.5%)	Zero		Zero	3 (10.7%)	2 (2.2%)	Zero	3 (1.7%)	2 (3.4%)	

* p value < 0.05.

## Discussion

A healthy lifestyle is incomplete without good eating habits. Vitamin A deficiency, iron deficiency anemia, and excess body weight are just a few of the health problems that young people face that can be avoided with proper nutrition. Eating habits, physical activity and smoking can also have a long-term impact on one's health. Unhealthy eating patterns, such as skipping breakfast and snacking foods high in carbohydrate and fat, are the leading causes of nutritional deficiencies. Heart disease, obesity, atherosclerosis, osteoporosis, and malignancies are all chronic diseases that can be aggravated by poor nutrition (31).

The current study findings showed a low daily frequency consumption of vegetables and fruits. It's varied from 16.07% of daily vegetable consumption to 11.23% of daily fruits consumption. More than 10% of all students reported no consumption of fruits and vegetables. A low prevalence of regular consumption of fruits and vegetables by undergraduates was reported in several studies conducted in various countries (33–38). Only 14% of 863 students in a recent study conducted at the University of Acre in Brazil's northern region consumed fruits and vegetables on a regular basis (38). The prevalence of inadequate fruit and vegetable intake among Turkish college students was 66.1 percent for men and 63.1% for women (36). Greek students' eating habits were also found to be particularly poor in terms of fruit and vegetable consumption, with 68.1% of men and 53.9% of women consuming fiber at levels below international guidelines (36). In addition, the current study revealed that when compared to students who were not stressed or mildly stressed during the previous month, those who were severely stressed did not eat any vegetables or fruits. Thus stress can act as factor which can influence the eating behaviors and negatively impact the students' health. These findings are consistent with the another study conducted in Saudi Arabia by Aljaber et al. (39), who reported that students with high levels of stress eat unhealthier foods than students with low levels of stress. A previous study conducted among Saudi Medical Students at University of Dammam, Saudi Arabia revealed that majority of the students (91.3%) were consuming fast foods, and were aware of the advantages of fruits and vegetables and the drawbacks of soft drinks, but they nevertheless tend to drink more soft drinks and consume less vegetables and fruits. And 65% of the male medical students did not routinely engage in physical activity (9). Similarly, another study conducted among Mutah University students in Jordan revealed a high prevalence of unhealthy eating behaviors with 59% of participants engaged in fast-food consumption ≥2 times per week (40).

The results of our study will serve as a basic documentation for regional health authorities and university managers to implement an intervention program to improve healthy eating habits and healthy lifestyle among students at Najran University. The benefits of such kind of nutrition interventions were shown elsewhere. For example, according to a Brazilian study, nutrition education initiatives on fruit and vegetable consumption that combined information and motivation were successful in impoverished environments (41). The actions included spreading awareness about the benefits of eating fruits and vegetables for good health and improving skills for incorporating them into daily eating. It implies that students would benefit from nutritional education programs.

Another important finding of the study showed a relatively high prevalence of smoking 25.77% among Najran university male students. This result was based on the self-reporting of students without any invasive investigation to identify objectively the smoking status. Otherwise, to overcome this limitation more precautions were taken during the study by using anonymous questionnaires and informing the students about the confidentiality of the results.

Similar range of prevalence (37%) were observed in Jeddah, in a study conducted among secondary school male students (42). Palestine (52.7%), Jordan (56.9%), and Egypt (61.2%) had the highest male smoking rates, according to a systemic review conducted in Arab countries (43). A prospective cohort multi-center international study of university students showed a high smoking prevalence among both male (34%) and female (27%) students. The highest prevalence was found in south European countries reaching 44% among male students, a lower prevalence was reported in Western Europe University male students about 31% (44).

Our study also showed a low level of awareness regarding health risks of smoking among Saudi male university students. Similar findings also revealed in another study conducted in Eastern Saudi secondary school adolescents, which showed that the prevalence of current smokers was 21.7% (45). Similarly, a research conducted in Botswana, South Africa, revealed that 10% of students were active tobacco smokers, with 29% of respondents having tried smoking (46). Another study conducted in Jordan revealed that the students started smoking to cope with course and exam stress. Students appreciated the feeling and flavor of cigarettes, especially with friends, and reported daily smoking of 10–40 cigarettes (47).

Anti-smoking education that does not include a discussion of mortality linked to smoking habits is ineffective. In fact, many international studies have shown that people who are aware of the mortality risks associated with smoking habits smoke less than those who believe smoking has reversible consequences (48). It was reported in other studies that students had personal justification and compensatory behavior to continue their smoking and to be protected from blame from themselves or from others (49). More behavioral studies are needed in the future to investigate these complex aspects.

Another important aspect of this study was determining the students' physical activity levels. This study revealed that 32.15% of participants were not practicing a 30 min of physical activity during 5 days per week, which is a little lower than the 45.8% of college students in a Saudi Arabian study (50). Our findings are consistent with previous research, which found that ~26.4% of university students in a Lebanese study engaged in physical activity (51). Physical inactivity was observed in approximately one-third of Chinese and Brazilian college students (52, 53). According to Makrides et al. (54), nearly half of Canadian university students exercise three or more times each week. A prior study in the United States (55) discovered that just 39% of students exercised three or more times per week. According to another American research, 47% of college students do not participate in intense exercise, and 17% were not physically active (56). According to the National College Health Risk Behavior Survey (NCHRBS) in the United States, 42% of the participants engaged in strenuous physical activity at least three times per week, whereas an additional 20% engaged in moderate—intensity activity (57). According to Staten et al., 41 and 39% of college students were moderately and vigorously physically active, respectively (58). Government statistics indicate that in many regions of the world, at least a quarter of all youths are physically inactive (59). The incidence of lack of activity in recreational time among college students in 23 countries varied with economic development and cultural factors, averaging 30% (central and eastern Europe), 23% (northwestern Europe and the United States), 42% (Pacific Asia), 39% (Mediterranean), and 44% (emerging economies) (60). This variation in physical inactivity levels between countries tends to reflect economic and social development, urbanization, and technological excellence. In the current study, 47.57% of students were physically active, of which 17.62% were engaged in daily 30 mins' physical activity for more than 6 months, which reflects the consistency and focus of the students on maintaining their physical fitness and wellbeing.

Also, the students' perceptions and beliefs about the benefits of performing 30 mins of regular physical activity was assessed in this study. Majority of students stated that physical activity has one or more benefits, particularly in terms of health promotion and maintenance. These findings were in contrast to the findings of Haase et al. (60), who found that awareness about activity and health was lacking, with only 40–60% of people knowing that physical activity was linked to the risk of cardiovascular disease.

Physical activity at the university should be encouraged as a preventive measure against chronic diseases and to improve quality of life in adult and elderly life, given the importance of the college years as a transition from adolescence to adulthood. University students require explicit, practical guidelines for participating in physical activity and staying fit and healthy.

## Conclusion

To conclude, the study findings showed a low daily frequency consumption of vegetables and fruits. More than 10% of the students did not consume vegetables and fruits at all. Another important finding of the study showed a relatively high prevalence of smoking and a low level of awareness regarding health risks of smoking among Saudi male university students. In addition, a large number of male university students were physically inactive, and could benefit from a structured intervention program, motivating the college students to engage in more vigorous exercise programs.

### Recommendations

University years is a time when the large majority of college students are entirely liable for their own eating patterns. Healthy eating habits developed during this time of transition may persist into adulthood, reducing the risk of many chronic diseases later down the line. Long-term nutrition intervention strategies should target enhancing nutrition attitudes and knowledge toward healthy eating amongst university students. In addition to this, there is a pressing need to enforce a tobacco-smoking-reduction strategies among students. As part of these strategies, medical schools and residency training programs could include training, lectures, and educational materials about smoking health risks. Urgent interventions such as setting boundaries and limits on technology and gadgets at school and home and encouraging healthy eating and physical activity are required. Officials at the university should also consider giving students free time slots to engage in physical activities. Students may benefit from mandatory physical activity classes at least three times per week.

### Limitations of the study

There are a few limitations worth mentioning for this study. It was a single centered, observational study involving solely male students from the health sciences colleges of Najran University, Saudi Arabia. Hence the results from this study cannot be generalized to other colleges in Saudi Arabia. Apart from that, the participants' self-reporting was used to measure the lifestyle behaviors. Hence, data could be skewed due to over-reporting or under-reporting.

## Data availability statement

The original contributions presented in the study are included in the article/supplementary material, further inquiries can be directed to the corresponding author.

## Ethics statement

The studies involving human participants were reviewed and approved by Scientific Ethical Committee of Najran University (NU/2019/12/3). The patients/participants provided their written informed consent to participate in this study.

## Author contributions

The author confirms being the sole contributor of this work and has approved it for publication.

## Funding

The author is thankful to the Deanship of Scientific Research, Najran University, Najran, Saudi Arabia, for funding this research through Grant Research Code NU/RC/MRC/11/3.

## Conflict of interest

The author declares that the research was conducted in the absence of any commercial or financial relationships that could be construed as a potential conflict of interest.

## Publisher's note

All claims expressed in this article are solely those of the authors and do not necessarily represent those of their affiliated organizations, or those of the publisher, the editors and the reviewers. Any product that may be evaluated in this article, or claim that may be made by its manufacturer, is not guaranteed or endorsed by the publisher.
